# Genetic and antigenic characterisation of serotype A FMD viruses from East Africa to select new vaccine strains

**DOI:** 10.1016/j.vaccine.2014.08.033

**Published:** 2014-10-07

**Authors:** Fufa D. Bari, Satya Parida, Tesfaalem Tekleghiorghis, Aldo Dekker, Abraham Sangula, Richard Reeve, Daniel T. Haydon, David J. Paton, Mana Mahapatra

**Affiliations:** a The Pirbright Institute, Pirbright Laboratory, Ash Road, Woking, Surrey GU24 0NF, UK; b Central Veterinary Institute, Part of Wageningen UR, Lelystad, The Netherlands; c Foot-and-Mouth Disease Laboratory, Embakasi, Nairobi, Kenya; d Boyd Orr Centre for Population and Ecosystem Health, Institute of Biodiversity, Animal Health and Comparative Medicine, College of Medical, Veterinary and Life Sciences, University of Glasgow, Glasgow G12 8QQ, UK

**Keywords:** FMD virus, Antigenic sites, Capsid sequence, Neutralising epitopes, Polyclonal antibodies, Vaccine strain selection

## Abstract

Vaccine strain selection for emerging foot-and-mouth disease virus (FMDV) outbreaks in enzootic countries can be addressed through antigenic and genetic characterisation of recently circulating viruses. A total of 56 serotype A FMDVs isolated between 1998 and 2012, from Central, East and North African countries were characterised antigenically by virus neutralisation test using antisera to three existing and four candidate vaccine strains and, genetically by characterising the full capsid sequence data. A Bayesian analysis of the capsid sequence data revealed the viruses to be of either African or Asian topotypes with subdivision of the African topotype viruses into four genotypes (Genotypes I, II, IV and VII). The existing vaccine strains were found to be least cross-reactive (good matches observed for only 5.4–46.4% of the sampled viruses). Three bovine antisera, raised against A-EA-2007, A-EA-1981 and A-EA-1984 viruses, exhibited broad cross-neutralisation, towards more than 85% of the circulating viruses. Of the three vaccines, A-EA-2007 was the best showing more than 90% *in-vitro* cross-protection, as well as being the most recent amongst the vaccine strains used in this study. It therefore appears antigenically suitable as a vaccine strain to be used in the region in FMD control programmes.

## 1 Introduction

Foot-and-mouth disease (FMD) causes serious production losses and has an enormous impact on trade. It is costly and difficult to control because of the diversity of the viruses involved, the multiple host species affected (both domestic and over 30 wildlife animal species) and the speed and different routes of transmission. It is caused by FMD virus (FMDV), a small non-enveloped RNA virus belonging to the genus *Aphthovirus* in the family *Picornaviridae*. The virus exists as seven immunologically distinct serotypes: O, A, C, Asia 1, Southern African Territory (SAT)-1, SAT-2 and SAT-3. Each serotype has a spectrum of antigenically distinct subtypes due to a high mutation rate [1]. The viral genome is about 8.3 kb long and enclosed in a protein capsid. The capsid comprises 60 copies each of the four structural proteins (VP1-VP4); the VP1-3 proteins are located on the surface, while VP4 is internal.

All FMDV serotypes produce a clinically indistinguishable disease but immunity to one serotype does not confer protection against another due to the antigenic diversity. The role of humoral antibodies as the principal component of FMD vaccine-induced protection is well established [2]. Traditionally, monoclonal antibody (mAb) resistant (mar) mutant studies and sequencing of their capsids have been used to identify critical amino acid (aa) residues for neutralisation [3], [4], [5], [6], [7], [8]. There are four known neutralising antigenic sites located on the three exposed capsid proteins of serotype A. Site 1 (G-H loop of VP1) is linear and trypsin-sensitive, whereas other sites are conformational and trypsin-resistant [5]. Crystallographic studies have identified that most neutralising epitopes have been found on surface oriented interconnecting loops between structural elements [9]. Mutation in an interconnecting loop may also cause distant effects by perturbation of loop stability [10]. The location of antibody binding sites (epitopes) or escape from binding can also be inferred from correlating the antibody cross-reactivity of viruses to their capsid sequence similarities [11]. Epitopes can also be predicted, in the absence of antibody recognition data, using different epitope prediction programmes using viral crystal structure [12]. However, there are no reports for analysis of epitopes or vaccine strain selection studies using serotype A isolates originating from East Africa.

Most FMD outbreaks in East Africa have been caused by serotype O, followed by serotype A and SAT-2 [13], [14], [15]. The serotype A viruses are present in all areas of the world where FMD has been reported and are diverse both antigenically and genetically. More than 32 subtypes [16] and 26 genotypes of serotype A FMDV have been reported [17]. Control of FMD mainly depends on the availability of matching vaccines that can be selected based on three criteria: epidemiological information, phylogeny of the gene sequence for evolutionary analysis and serological cross-reactivity of bovine post-vaccinal serum (bvs) with circulating viruses [18], [19]. Mono-, bi- and quadri-valent vaccines are currently in use in East African countries for FMD control [20], [21], [22]. These vaccines are mainly produced in vaccine production plants located in Ethiopia and Kenya using relatively historic viruses and regular vaccine matching tests to select the best vaccine for use in the region are rarely carried out. Hence, the existing vaccines may not provide optimal protection against recently circulating FMD viruses. This study was, therefore, designed to characterise recently circulating FMD viruses in the region both antigenically and genetically and recommend matching vaccine strains for use in FMD control program in East African countries.

## 2 Materials and methods

### 2.1 Cells and viruses

Fifty-six serotype A viruses from Africa submitted to the World Reference Laboratory for FMD (WRLFMD) at Pirbright were used in this study. These viruses were from five East African countries, Ethiopia (*n* = 8), Eritrea (*n* = 9), Sudan (*n* = 6), Kenya (*n* = 6), Tanzania (*n* = 7) and from three neighbouring countries: Democratic Republic of Congo (COD, *n* = 5), Egypt (*n* = 10) and Libya (*n* = 5). These samples are known to have been derived from cattle epithelial tissues except eight viruses from Egypt and one virus from Kenya where the host species is not known (Supplementary Table 1). All the samples were initially grown in primary bovine thyroid cells (BTY) with subsequent passage in either BHK-21 or IB-RS2 cells. The virus stocks were prepared by infecting cell monolayers and stored at −70 °C until use. Viruses are named according to a three letter code for the country of origin followed by the isolate number and the year of isolation, *e.g.* A-COD-02-2011. Candidate vaccine strains are designated by a two letter code for East Africa followed by the year of isolation, *e.g.* A-EA-2005.

### 2.2 Polyclonal sera

Seven anti-FMDV bovine post-vaccinal sera were used in the study. Two were against the two existing vaccine strains, A-KEN-05-1980 and A-ETH-06-2000 raised in Kenya and Ethiopia [21], respectively, by administering the commercially prepared vaccine. The animals vaccinated with A-KEN-05-1980 were bled on 21 day following vaccination. The animals vaccinated with A-ETH-06-2000 received a boost on 21-day post-vaccination and bled one week later. The rest five bvs were raised in cattle against one existing vaccine strain (A-ERI-1998) and four candidate vaccine strains (A-EA-1981, A-EA-1984, A-EA-2005 and A-EA-2007) following the method previously described [23]. The candidate vaccine strains were selected taking into account the genotypes currently circulating in the region. For each antigen, sera from four or five animals were pooled for use in the neutralisation test. The homologous neutralising antibody titres of each pooled serum are presented in Table 1a.

**Table 1a:** Homologous neutralisation serum titres of the seven bovine post-vaccinate sera.

Vaccine	Number of individual serum samples pooled	Mean serum titre (log_10_)
A-EA-2007	5	2.54
A-ERI-1998	5	2.49
A-EA-1984	5	2.40
A-EA-2005	4	2.36
A-KEN-05-1980	4	2.13
A-EA-1981	5	2.09
A-ETH-06-2000	5	1.99

The vaccines are arranged in decreasing order of serum titre.

### 2.3 Two-dimensional micro-neutralisation test (2D-VNT)

The 2D-VNT test was carried out using the pooled post-vaccination bovine sera according to Rweyemamu and colleagues. [24]. Antibody titres were calculated from regression data as the log_10_ reciprocal antibody dilution required for 50% neutralisation of 100 tissue culture infective units of virus (log_10_SN_50_/100 TCID_50_). The antigenic relationship of viruses is given by the ratio: ‘*r*_1_’ = neutralising antibody titre against the heterologous virus/neutralising antibody titre against the homologous virus. The significance of differences between ‘*r*_1_-values’ obtained by the polyclonal antiserum was evaluated according to standard criteria [25].

### 2.4 Nucleotide sequencing and analysis of the sequence data

The sequences of the entire capsid coding region (P1) of the viruses were generated. RNA extraction from the cell culture grown viruses, reverse transcription (RT), polymerase chain reaction (PCR) to amplify the P1 region, sequencing, sequence analysis and assembling, and alignment were performed as described previously [26]. MEGA 5 [27] was used to determine nucleotide and aa variations. The aa variability of the capsid coding region of the type A viruses were determined as described by Valdar [28].

### 2.5 Genetic characterisation

The aligned, complete P1 nucleotide sequences were used to determine the most suitable nucleotide substitution model using jModelTest [29] and MEGA [27] resulting in the selection of a General time reversal (GTR) model with a combination of gamma distribution and proportion of invariant sites (GTR + G + I). Then, Bayesian analysis was performed using the BEAST software package v1.5.4 [30]. In BEAUti v1.5.4, the ages of the viruses were defined by the date of sample collection and the analysis used GTR + G + I model to describe rate heterogeneity among sites. Variations in substitution rate among branches were evaluated by comparing four different clocks in BEAST. The maximum clade credibility (MCC) phylogenetic tree was inferred using the Bayesian Markov Chain Monte Carlo (MCMC) method. Then, a Bayes factor analysis in TRACER version 1.5 [31] was used to determine the best-fit model that resulted in the selection of an uncorrelated exponential relaxed molecular clock. The tree was obtained using the Tree Annotator program in BEAST and the evolutionary trees were viewed in FigTree program 1.3.1.

### 2.6 Statistical analysis

The relationship between predicted protection (*r*_1_-value ≥0.3) and changes in aa was analysed using a general linear model (GLM) with binomial error distribution. For this, a binomial variable ‘protected/not protected’ was created based on the estimated *r*_1_-values ≥0.3 (protected), which was used as the response variable. Summaries of the aa count differences between the query sequence of the vaccine strain and those of the field viruses were used as independent variables using either entire P1 aa sequence and each of the different viral proteins (VP1-4), alone or in combination. Both variables were analysed independently in a univariate analysis and together in a multivariate analysis. The GLM modelling and analysis of the data was carried out using *R* [32].

## 3 Results and discussion

In FMD endemic settings, implementation of the progressive disease control pathway [13] requires vaccines that can protect against both circulating and emerging variants, regular vaccination campaigns, post-vaccination sero-monitoring and biosecurity measures in the form of livestock movement control. Therefore, selection of appropriate vaccine strains is an important element in implementing vaccination policies for the control of FMD. FMD is enzootic in East Africa, with outbreaks reported regularly [15], [33], [34], [35]. Although the region has two vaccine producing plants, there is little information available on the protective value of the supplied vaccines. The only report on vaccine strain selection in East Africa [21] was limited to a small selection of Ethiopian vaccines (two) and viruses (five). In addition, Kenya uses historic viruses such as A-KEN-05-1980 (A/K/5/80) and A-KEN-35-1980 (A/K/35/80) for vaccine production [22] and the vaccine matching tests are seldom carried out [15]. In these settings, where emergence of new variants is unpredictable, especially for serotype A FMDV, continuous serological and genetic characterisations of field viruses is needed to understand the cross-reactivity of existing vaccines and to trace patterns of viral spread.

### 3.1 Serological characterisation of serotype A FMD viruses

**Fig. 1:**
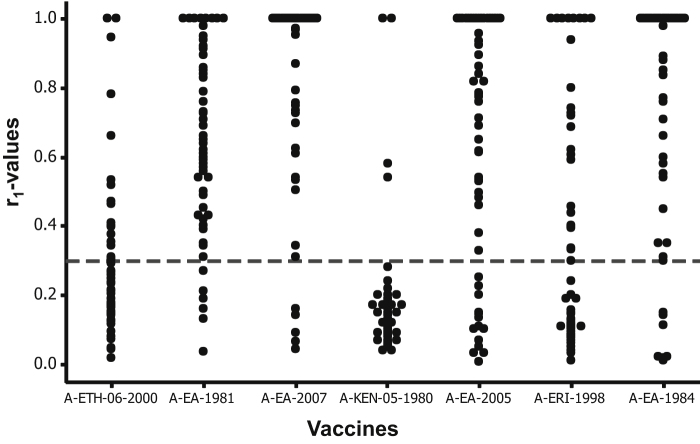
Antigenic relationship (*r*_1_) values of 56 East Africa type A isolates. The serological match (*r*_1_-values) of the seven vaccine strains is shown as black dots. The horizontal dotted line indicates the cut-off value of 0.3, above which the vaccine is considered to be a good match.

In this study, the ability of the three existing vaccine strains (A-ERI-1998, A-ETH-06-2000 and A-KEN-05-1980) and four putative candidate vaccine strains (A-EA-2007, A-EA-1984, A-EA-2005 and A-EA-1981) of serotype A FMDV to cross-protect (*in-vitro*) against the circulating viruses was measured by 2D VNT. The three existing vaccine strains were found to be least cross-reactive (*r*_1_-values ≥0.3 observed for only 5.4–46.4% of the sampled viruses) suggesting a poor suitability in the field, unless the low antigenic match can be compensated for by highly potent vaccine formulations [36]. However A-ETH-06-2000 and A-ERI-1998 exhibited good cross-reactivity against viruses within their respective genotypes and also the viruses from their respective countries (Table 1b). The viruses not neutralised by the seven bvs were the Asian topotype (A-Iran-2005 strain) viruses. The most broadly reactive antisera were A-EA-2007, A-EA-1981 and A-EA-1984 exhibiting 91.1%, 89.3% and 87.5% *in-vitro* protection, respectively, (Fig. 1 and Table 1b) and could be strong candidates to be developed as vaccine strains. However A-EA-1984 may not be suitable for the region as the A-Iran-05 like viruses circulating in Libya were not covered by this vaccine at all (Table 1b). There is evidence of incursion of the viruses circulating in the Middle East into African countries like Egypt and Libya because of animal trade between these countries [37]. Therefore these viruses may also be subjected for an antigenic match along with East African outbreak viruses, as these viruses may spread into East African countries because of unrestricted animal movement between African nations. Since developing and maintaining two vaccine strains for use along with the associated quality control and vaccine potency tests is not very attractive to vaccine manufacturers, it would be better to select a single strain, such as A-EA-2007 that showed broad cross-reactivity to the circulating strains of different genotypes and topotypes. A final decision would need to take account of other criteria, such as the virus yield in cell culture and the stability of the antigen produced.

**Table 1b:** Predicted *in vitro* protection provided by individual vaccine strains against field viruses per country.

Country of virus origin		Proportion of field viruses protected by each vaccine						
	Number of field viruses tested per country	A-ETH-06-2000^*, $^	A-EA-1981	A-EA-2007^$^	A-ERI-1998^*,#^	A-KEN-05-1980^*,£^	A-EA-2005^£^	A-EA-1984^#^
Ethiopia^$^	8	7/8	8/8	8/8	3/8	0/8	6/8	8/8
COD^£^	5	1/5	4/5	5/5	3/5	0/5	5/5	5/5
Kenya^£^	6	2/6	6/6	6/6	3/6	3/6	3/6	5/6
Tanzania^£^	7	2/7	6/7	7/7	1/7	0/7	7/7	7/7
Eritrea^#^	9	3/9	9/9	9/9	9/9	0/9	7/9	9/9
Sudan^#^	6	3/6	4/6	6/6	5/6	0/6	6/6	6/6
Egypt^$,#^	10	2/10	8/10	7/10	2/10	0/10	7/10	9/10
Libya	5	0/5	5/5	3/5	0/5	0/5	1/5	0/5
Total (% protection)	56	20/56 (35.7)	50/56 (89.3)	51/56 (91.1)	26/56 (46.4)	3/56 (5.4)	42/56 (75.0)	49/56 (87.5)

^*^ Commercial vaccine in use. Countries and vaccine viruses having the same superscript (^£^-genotype I, ^#^-genotype IV, ^$^-genotype VII) belong to the same genotype. Shaded boxes indicate best vaccines for each country. Where multiple vaccine strains provide equivalent expected protection, the best recommendation is for the one with the closest genetic relationship to the viruses circulating within the country.

### 3.2 Genetic characterisation of the serotype A viruses

The full capsid sequences of the 56 serotype A viruses generated in this study were 2205 nucleotides (nt) long. The viruses showed a total of 882 (40%) nt and 158 aa (21.5%) aa substitutions across P1 (Table 2a). Compared to the oldest virus A-KEN-05-1980 there was 0.2% (A-KEN-01-2003) to 23.7% (A-EGY-08-2011) nucleotide variation between these viruses. Analysis of the capsid amino acid sequences revealed 0.3% (A-KEN-01-2003) to 9.5% (A-EGY-08-2011) aa variation. Similarly, compared to the best vaccine virus, A-EA-2007, there were 3.3% (A-ETH-13-2009) to 25.2% (A-EGY-05-2011) nucleotide variation and the amino acid variation was found to be 0.1% (A-ETH-07-2008) to 8.6% (A-EGY-05-2011, A-EGY-01-2010, A-LIB-21-2009).

**Table 2a:** Percentage (%) nucleotide and amino acid variability in the capsid sequences of the serotype A FMD viruses.

Capsid region	Total no. of nt positions aligned	No. of variable nt	% nt variability	Total no. of aa positions aligned	No. of variable aa	% aa variability
VP1	633	295	46.6	211	71	33.65
VP2	654	251	38.38	218	38	17.43
VP3	663	250	37.71	221	41	18.55
VP4	255	86	33.73	85	8	9.41
P1	2205	882	40	735	158	21.5

nt: nucleotide, aa: amino acid, %: percent.

The analysis of the capsid aa residues of the type A viruses revealed a large number of sites across the capsid having 4–8 alternative aa (Table 2b). Notably, sequences for VP2-191 encoded eight different amino acids (A/N/D/Q/G/H/S/T) and exhibited nt changes at all the three positions within the codon (Table 2b) as did VP2-134 (A/N/E/Q/P/T/V) and VP1-197 (A/G/L/P/S/T/V) giving rise to seven alternative amino acids. Recently, residues VP1-197 and VP2-191 were predicted as epitopes for serotype A FMD viruses using various epitope prediction software [12]. VP2-191 has also been shown to be of antigenic significance in case of serotype O viruses [38]. VP2-134 is located adjacent to VP2-132, a known neutralising epitope in serotype A10 [6]. In addition, this residue has been reported to strongly influence the binding of neutralising antigenic site 2 mAbs in serotype O FMDV [39]. Therefore, these residues could be of antigenic significance in serotype A viruses which requires further investigation.

**Table 2b:** Capsid positions where multiple amino acid substitutions were observed. Numbers in parenthesis indicates number of isolates in which these changes were observed.

Viral protein	Number of alternative aa residues				
	4	5	6	7	8
VP2	71 (27),74 (27), 78 (27), 86 (32), 131 (6), 173 (31)	64 (12)	133 (26)	134 (38)	191 (41)
VP3	70 (29),139 (12)	220 (30)	71 (30)	–	–
VP1	24 (17), 96 (21), 101 (24), 110 (29), 139 (32), 140 (28), 142 (29), 148 (16), 149 (30), 194 (9)	44 (33), 141(29), 196 (21), 198 (31)	43 (24), 45 (33), 99 (40)	197 (32)	–

#### 3.2.1 Bayesian phylogenetic analysis

**Fig. 2:**
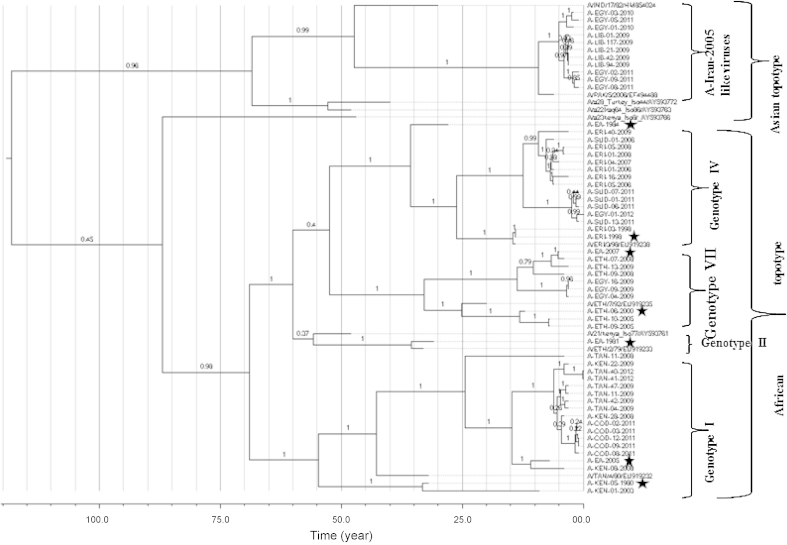
Bayesian phylogenetic tree of the FMDV serotype A isolates from East Africa and three neighbouring countries. The reference capsid sequences (*n* = 10) retrieved from Genbank are indicated by their respective accession numbers. The stars indicate the vaccine viruses.

Phylogenetically, the viruses were grouped into two topotypes (African and Asian) within serotype A FMDV. In East Africa, only four genotypes (I, II, IV, and VII; Fig. 2) of African topotype viruses were found to be circulating, along with four viruses from Egypt and five viruses from COD. Interestingly, all the viruses isolated from COD belong to genotype I (Fig. 2), similar to isolates from neighbouring countries such as Tanzania and Kenya, suggesting cross-border livestock movement and/or trade between these countries as observed in Uganda [40], Libya and Egypt [37].

A-EA-1981 virus was assigned to genotype II, however no further viruses of this genotype have been detected in the region since. The Asian topotype viruses (A-IRAN-2005 like viruses) were detected only in Egypt and Libya. These viruses were also detected in 2013 in Egypt and may still be circulating in the region. The scenario in Egypt is further complicated by circulation of two African genotypes (G IV and VII; Fig. 2) thereby making FMD control very difficult. The introduction of A-IRAN-2005 like viruses to Africa could be the result of trade between the Middle East and African countries [37].

#### 3.2.2 Rate of nucleotide substitution

BEAST analysis using selected models revealed that the mean rate of nucleotide substitution in the capsid coding region of the viruses (year of isolation 1964 to 2012) was estimated to be 3.09 × 10^−3^ substitution/site/year (95% HPD 2.02 × 10^−3^ to 4.16 × 10^−3^). This is lower than the rate reported for VP1 sequences of serotype A viruses [41] and that for P1 sequences of A-Iran-05 like viruses from the middle-East [26].

The mean estimate of the time of emergence for the most recent common ancestor was found to be about 128 years before the present (ybp) [95% highest posterior density (HPD): 69 to 212]. This compares to a previous estimate of about 178 ybp (in 1823) for the emergence of serotype A viruses [41]. According to our estimation, the common ancestor of East Africa serotype A viruses existed around 1926 (Fig. 2).

### 3.3 Amino acid variability of the capsid of the type A viruses from East Africa

**Fig. 3:**
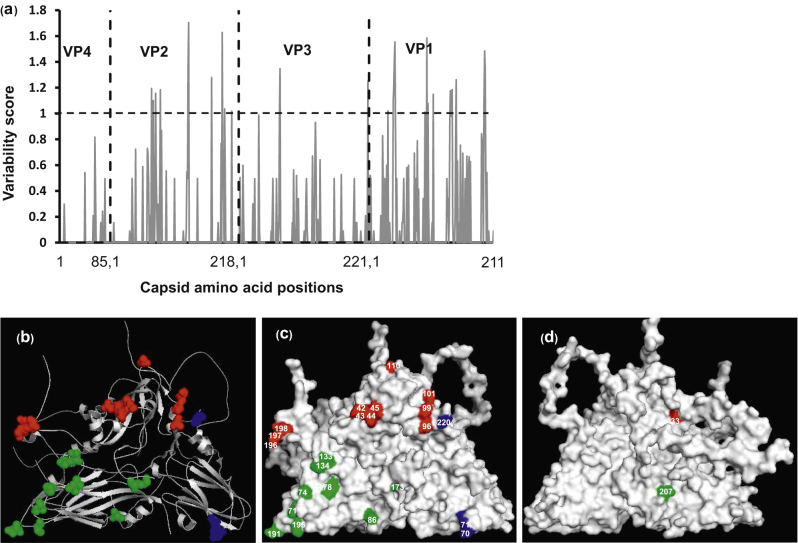
(a) Capsid amino acid variability of the type A viruses from East-Africa. The vertical black dotted line indicates gene junction. ((b)–(d)) 3D structure of Type A protomer of A10_61_ (1ZBE) showing highly variable capsid amino acid residue (with a score ≥1) highlighted. VP1 residues (red), VP2 residues (green) and VP3 residues (blue); (b) cartoon; (c) external surface (d) internal surface. (For interpretation of the references to color in this figure legend, the reader is referred to the web version of this article.)

Analysis of the variability of the capsid amino acids of the type A viruses from East-Africa revealed VP4 to be highly conserved and VP1 to be highly variable (Table 2a and Fig. 3a); similar to earlier reports on type A viruses from the Middle East [26]. The residues with a score greater than 1.0 (16 in VP1, 10 in VP2 and 3 in VP3) are shown in Fig. 3a indicating that more than 50% of the residues with a high variability score are present in VP1. All but two (VP1-33 and VP2-207) of these residues were found to be surface exposed (Fig. 3b–d).

### 3.4 Correlating cross-reactivity with capsid amino acid changes

The association between the numbers of aa changes and the serological reactivity (expressed as probability of protection; *r*_1_-value ≥0.3) between vaccine and virus strain pairs was assessed using a GLM model. This analysis was done for the whole P1 region and for the four individual structural proteins (VP1-4) either alone or in combinations. First, a univariate analysis was carried out, which showed that the number of changes in the P1 and VP4 proteins did not correlate to *in-vitro* cross-protection, whereas a link was evident for the three surface-exposed proteins (VP1-3), with VP3 showing the strongest association (*P* < 0.001). A subsequent multivariate analysis to evaluate the three different VP regions and their interactions did not identify any significant interactions. Changes in VP3 and VP2 showed a significant (negative) effect on the probability of protection; the higher the number of changes the lower the probability of protection (Supplementary Table 2). The absence of a relationship between predicted protection of vaccines and changes in capsid aa of field viruses observed in our analysis is in keeping with other evidence that neutralisation is governed by key (mutant-) capsid aa residues, and probably by residue interactions, rather than overall residue changes [10]. However, the observation of a relationship between predicted protection and the substitution of aa in VP3 is interesting. Assessing the contribution of specific substitutions to predicted cross-protection requires more advanced analytical approaches and manipulation of selected aa residues using reverse genetics approaches.

The multivariate analysis also allowed a comparison of the predicted level of cross-protection provided by each of the commercial and candidate vaccine strains used in this study. A-EA-2007, A-EA-1984 and A-EA-1981 exhibited significantly higher expected protection with A-EA-2007 exhibiting the highest odds value (Table 3). A-ETH-06-2000 was not significantly different from A-ERI-1998, while A-KEN-05-1980 was significantly less protective than A-ERI-1998. The vaccines (A-ETH-06-2000 and A-KEN-05-1980) showing the lowest *in-vitro* cross-protection based on *r*_1_-values (Fig. 1) also showed the lowest odd values (Table 3).

**Table 3:** Odds of protection of all vaccines with reference to vaccine strain A-ERI-1998 was used as reference vaccine for model selection as it is the best amongst the commercially available vaccines.

Vaccine	Odds value	95% confidence interval	*P*-value
A-ERI-1998	1	–	–
A-EA-2007	36.17	11.57–135.02	*P* < 0.001
A-EA-1984	29.85	9.54–109.92	*P* < 0.001
A-EA-1981	16.25	5.56–54.90	*P* < 0.001
A-EA-2005	8.66	3.43–23.32	*P* < 0.001
A-ETH-06-2000	0.73	0.31–1.70	*P* > 0.5
A-KEN-05-1980	0.06	0.01–0.21	*P* < 0.001

In conclusion, two topotypes (African and Asian) of the type A viruses were detected in East Africa; of the native African topotype three genotypes are currently circulating in the region. We have recommended different vaccines for the different genotypes based on their serological cross-reactivity and genetic relationship. A-EA-2007 has broader cross-reactivity and is also a recent isolate; therefore, is recommended as a potential vaccine strain candidate to be used in FMD control programs in East Africa, subject to good growth and stability characteristics and *in vivo* evaluation in the target host.
