# Supplementary material for: Genetic and antigenic characterisation of serotype A FMD viruses from East Africa to select new vaccine strains
Source: Vaccine. 2014 Oct 7;32(44):5794–800. doi: 10.1016/j.vaccine.2014.08.033 (PMC4194315; doi:10.1016/j.vaccine.2014.08.033)
Supplement: Supplementary file 1 [file mmc1.pdf]

Supplementary Table 1 List of viruses used in this study.

No	Virus name	Topotype	Genotype	Country, Region	Host	Date collected	Accession No.
1	A-COD-02-2011	African	I	DRC, Central Africa	Cattle	01/02/2011	This study
2	A-COD-03-2011	African	I	DRC, Central Africa	Cattle	02/02/2011	This study
3	A-COD-08-2011	African	I	DRC, Central Africa	Cattle	02/02/2011	This study
4	A-COD-09-2011	African	I	DRC, Central Africa	Cattle	03/02/2011	This study
5	A-COD-12-2011	African	I	DRC, Central Africa	Cattle	03/02/2011	This study
6	A-ETH-06-2000	African	VII	Ethiopia, East Africa	NK	2000	This study
7	A-ETH-09-2005	African	VII	Ethiopia, East Africa	Cattle	27/12/2000	This study
8	A-ETH-10-2005	African	VII	Ethiopia, East Africa	Cattle	11/01/2002	This study
9	A-EA-1981	African	II	Ethiopia, East Africa	Cattle	1981	This study
10	A-EA-2007	African	VII	Ethiopia, East Africa	Cattle	07/12/2007	This study
11	A-ETH-07-2008	African	VII	Ethiopia, East Africa	Cattle	08/01/2008	This study
12	A-ETH-09-2008	African	VII	Ethiopia, East Africa	Cattle	01/08/2008	This study
13	A-ETH-13-2009	African	VII	Ethiopia, East Africa	Cattle	14/02/2009	This study
14	A-ERI-1998	African	IV	Eritrea, East Africa	Cattle	15/01/1998	This study
15	A-ERI-03-1998	African	IV	Eritrea, East Africa	Cattle	15/01/1998	EU919238
16	A-ERI-05-2006	African	IV	Eritrea, East Africa	Cattle	01/01/2006	This study
17	A-ERI-04-2007	African	IV	Eritrea, East Africa	Cattle	14/02/2007	This study
18	A-ERI-01-2008	African	IV	Eritrea, East Africa	Cattle	04/01/2008	This study
19	A-ERI-05-2008	African	IV	Eritrea, East Africa	Cattle	16/12/2008	This study
20	A-ERI-01-2006	African	IV	Eritrea, East Africa	Cattle	15/12/2006	This study
21	A-ERI-16-2009	African	IV	Eritrea,East Africa	Cattle	09/10/2009	This study
22	A-ERI-40-2009	African	IV	Eritrea, East Africa	Cattle	19/10/2009	This study
23	A-EGY-04-2009	African	VII	Egypt, North Africa	Cattle	01/01/2009	This study
24	A-EGY-09-2009	African	I	Egypt, North Africa	Cattle	08/02/2009	This study
25	A-EGY-16-2009	African	VII	Egypt, North Africa	NK	12/02/2009	This study
26	A-EGY-01-2010	Asian	A-Iran-2005	Egypt, North Africa	NK	14/10/2010	This study
27	A-EGY-03-2010	Asian	A-Iran-2005	Egypt, North Africa	NK	15/10/2010	This study
28	A-EGY-02-2011	Asian	A-Iran-2005	Egypt, North Africa	NK	09/01/2011	This study
29	A-EGY-05-2011	Asian	A-Iran-2005	Egypt, North Africa	NK	11/04/2011	This study
30	A-EGY-08-2011	Asian	A-Iran-2005	Egypt, North Africa	NK	19/05/2011	This study
31	A-EGY-09-2011	Asian	A-Iran-2005	Egypt, North Africa	NK	14/06/2011	This study
32	A-EGY-01-2012	African	IV	Egypt, North Africa	NK	08/02/2012	This study
33	A-KEN-05-1980	African	I	Kenya, East Africa	NK	1980?	This study
34	A-EA-2005	African	I	Kenya, East Africa	Cattle	15/07/2005	This study
35	A-KEN-01-2003	African	I	Kenya, East Africa	Cattle	18/11/2003	This study
36	A-KEN-08-2008	African	I	Kenya, East Africa	Cattle	19/02/2008	This study
37	A-KEN-28-2008	African	I	Kenya, East Africa	Cattle	01/08/2008	This study
38	A-KEN -22-2009	African	I	Kenya, East Africa	Cattle	01/03/2009	This study
39	A-LIB-01-2009	Asian	A-Iran-2005	Libya, North Africa	Cattle	20/02/2009	This study
40	A-LIB-21-2009	Asian	A-Iran-2005	Libya, North Africa	Cattle	20/02/2009	This study
41	A-LIB-42-2009	Asian	A-Iran-2005	Libya, North Africa	Cattle	23/02/2009	This study
42	A-LIB-94-2009	Asian	A-Iran-2005	Libya, North Africa	Cattle	01/01/2009	This study
43	A-LIB-117-2009	Asian	A-Iran-2005	Libya, North Africa	Cattle	06/03/2009	This study
44	A-SUD-01-2006	African	IV	Sudan, East Africa	Cattle	09/11/2006	This study
45	A-EA-1984	African	IV	Sudan, East Africa	Cattle	04/11/1984	This study
46	A-SUD-01-2011	African	IV	Sudan, East Africa	Cattle	28/02/2011	This study
47	A-SUD-06-2011	African	IV	Sudan, East Africa	Catle	01/03/2011	This study
48	A-SUD-07-2011	African	IV	Sudan, East Africa	Catle	01/03/2011	This study
49	A-SUD-13-2011	African	IV	Sudan, East Africa	Catle	03/03/2011	This study
50	A-TAN-11-2008	African	I	Tanzania, East Africa	Cattle	01/08/2008	This study
51	A-TAN-04-2009	African	I	Tanzania, East Africa	Cattle	01/05/2009	This study
52	A-TAN-11-2009	African	I	Tanzania, East Africa	Cattle	21/06/2009	This study

53	A-TAN-42-2009	African	I	Tanzania, East Africa	Cattle	07/09/2009	This study
54	A-TAN-47-2009	African	I	Tanzania, East Africa	Cattle	01/11/2009	This study
55	A-TAN-40-2012	African	I	Tanzania, East Africa	Cattle	01/06/2012	This study
56	A-TAN-41-2012	African	I	Tanzania, East Africa	Cattle	01/06/2012	This study
57	A/21Kena iso77	African	I	Kenya, East Africa	NK	1964	AY593761
58	A/a23Kenya iso8	African	I	Kenya, East Africa	NK	1965	AY593766
59	A/TAN/4/80	African	I	Tanzania, East Africa	NK	1980	EU919232
60	A/ETH/7/92	African	VII	Ethiopia, East Africa	NK	1992	EU919235
61	A/ETH/7/79	African	VII	Ethiopia, East Africa	NK	1979	EU919233
62	A/IND/17/82	Asian	NA	India	Cattle	1982	HM854024
63	A/PAK/5/2006	Asian	NA	Pakistan	Cattle	2006	EF494488
64	A/a28_Turkey_ios44	Asian	NA	Turkey	NK	1972	AY593772
65	A/a22iraq64_iso86	Asian	NA	Iraq	NK	1964	AY593763

NA- not assigned, NK-not known

Supplementary Table 2. Results of the multivariate analysis.

Parameter	Estimate*	Std. Error	z value	Pr(>|z|)	Significance
Intercept	3.04	0.63	4.85	0.00	***
A-EA-2007	3.59	0.62	5.78	0.00	***
A-EA-1981	2.79	0.58	4.82	0.00	***
A-EA-2005	2.16	0.49	4.43	0.00	***
A-EA-1984	3.40	0.62	5.48	0.00	***
A-ETH-06-2000	-0.32	0.44	-0.74	0.46	NS
A-KEN-05-1980	-2.80	0.70	-4.00	0.00	***
VP3	-0.17	0.05	-3.09	0.00	**
VP2	-0.13	0.05	-2.50	0.01	*

*Estimates are presented as log odd ratios, NS-not significant.
